# The Precautionary Principle and Expert Disagreement

**DOI:** 10.1007/s10670-021-00457-y

**Published:** 2021-09-20

**Authors:** Lee Elkin

**Affiliations:** Institute of Philosophy/INCET, Jagiellonian University in Kraków, Grodzka 52, 31-044 Kraków, Poland

## Abstract

The Precautionary Principle is typically construed as a conservative decision rule aimed at preventing harm. But Martin Peterson (JME 33: 5–10, 2007; The ethics of technology: A geometric analysis of five moral principles, Oxford University Press, Oxford, 2017) has argued that the principle is better understood as an epistemic rule, guiding decision-makers in forming beliefs rather than choosing among possible acts. On the epistemic view, he claims there is a principle concerning expert disagreement underlying precautionary-based reasoning called the ecumenical principle: all expert views should be considered in a precautionary appraisal, not just those that are the most prominent or influential. In articulating the doxastic commitments of decision-makers under this constraint, Peterson precludes any probabilistic rule that might result in combining expert opinions. For combined or consensus probabilities are likely to provide decision-makers with information that is more precise than warranted. Contra Peterson, I argue that upon adopting a broader conception of probability, there is a probabilistic rule, under which expert opinions are combined, that is immune to his criticism and better represents the ecumenical principle.

## Introduction

To mitigate the risk of harm from a given activity, policymakers should institute precautionary measures, even in case of scientific uncertainty. That is the idea underlying the so-called Precautionary Principle (PP). However, there is little consensus on how exactly the PP is defined (see Boyer-Kassem, 2017). Although there is no universally accepted formulation of the principle, the PP is typically construed as a constraint on decision-making under uncertainty. 1

But Martin Peterson (2006) has challenged the received view with an impossibility result “showing that no version of the precautionary principle can be reasonably applied to decisions that may lead to fatal outcomes” (Peterson, 2007, p. 6). Despite dealing a significant blow to the PP, Peterson (2007, 2017) suggests that the principle can be preserved by interpreting it as an epistemic rule, guiding decision-makers in forming doxastic attitudes, absent scientific certainty. In other words, the PP should instead be construed as a rule for advising decision-makers on what to *believe* in the face of scientific uncertainty, not choosing among possible acts.

On the epistemic view, Peterson (2007) claims that the PP is grounded by three principles. They are a *preference for false positives*, i.e., prioritize null hypothesis statistical significance testing in scientific research. The *ecumenical principle*, i.e., all expert views should be considered in a precautionary appraisal, not just those that are the most prominent or influential. The *principle of non-monotonicity*, i.e., more is not always better. Granting that the PP might indeed be better understood as an epistemic rule grounded by the latter principles, at least in scientifically relevant matters, my focus in this paper is on the ecumenical principle.

In carefully articulating it, Peterson characterizes the doxastic commitments of decision-makers in terms of qualitative, full beliefs, which preclude decision-makers from forming probabilistic degrees of belief or credences in response to expert disagreement. His reason is that if the doxastic commitments of decision-makers are interpreted probabilistically, the principle might be taken as a rule for aggregating opinions, such as weighted averaging, to form a consensus view on behalf of experts. But combined or consensus probabilities are likely to overstate the evidence by providing decision-makers with information more precise than warranted. However, Peterson’s reasoning is short-sighted and has led him to advance an inadequate account of the ecumenical principle, considering that expert opinions concerning scientific hypotheses and theories often vary in strength, and so should those of decision-makers.

In this paper, I argue that upon accepting that beliefs vary in strength and that they might also be *imprecise*, a feasible imprecise probabilistic representation of the doxastic commitments of decision-makers can be given, under which conflicting expert judgments are combined. I will show that the proposed account of the ecumenical principle is partially consistent with the doxastic constraints imposed by Peterson, but satisfying weaker versions of the constraints only is fortunate, as the proposed account avoids an inconsistency his account gives rise to due to its overly permissive nature. Furthermore, the reader will find that the imprecise probabilistic representation is not subject to Peterson’s criticism that combined probabilities are prone to overstating the evidence and that in addition, it avoids an opposite concern that his account is susceptible to, namely, understating the evidence. The proposed account therefore enjoys several advantages over Peterson’s and better represents the ecumenical principle.

## The Ecumenical Principle

Disagreement occurs on practically every matter, often leading to epistemic conflict. While some might have the intuition that disagreement is a negative feature of discourse, especially in the context of scientific research, such an intuition has been shown to be false. Under certain conditions, disagreement can improve the accuracy of collective judgments via the ‘wisdom of crowds’ (Surowiecki, 2005), promote different perspectives (Harding, 1991; Longino, 1990), and prompt reflective evaluation resulting in epistemic humility (Elga, 2007).

Although disagreement yields some epistemic benefits, it can hinder progress by inducing further uncertainty. It is unsurprising then why non-expert decision-makers, such as policymakers, often have difficulty devising a plan of action when the evidence made available to them by an expert community is conflicting. But even so, decision-makers must continue moving towards meeting their objectives. How, though, should they proceed when confronted with conflicting expert opinions? Cue the ecumenical principle.

In cases where experts disagree, it is often difficult for the decision maker to take this disagreement into account in a reasonable way. In many cases, one simply has to decide which expert appears to be most trustworthy. Arguably, this is something that could be questioned from an epistemic point of view. According to the ecumenical principle, all expert views should be considered in a precautionary appraisal, not only the views put forward by the most prominent or influential expert. (Peterson, 2007, p. 8)

Put more succinctly, the ecumenical principle might be defined as follows.

Ecumenical Principle (EP). A theory or proposition is precautionarily appraised if and only if all expert views on the matter are taken into account, not just those of the most prominent or influential experts.

Under this generic formulation of the principle, it follows that decision-makers are compelled to respect the views of all experts. However, the principle as defined lacks specifics on what beliefs decision-makers should arrive at as a result.

Peterson addresses the latter deficiency by characterizing the doxastic commitments of decision-makers through the following constraints.

(OB)ligation: it is obligatory to believe a proposition *X* if and only if every expert believes *X*;

(PER)mission: it is permissible to believe a proposition *X* if and only if at least some experts believe *X*;

(PRO)hibition: it is forbidden to believe a proposition *X* if and only if no expert believes *X*. (2007, p. 9)

Notice that the doxastic commitments of decision-makers on Peterson’s view are restricted to qualitative, full beliefs. This restriction engenders an obvious practical advantage. In particular, when choosing among possible acts, decision-makers only need to decide on the basis of their adopted full beliefs rather than taking on a more arduous task of identifying the optimal act(s) through some complex decision calculus.

However, it is not obvious that the doxastic commitments of decision-makers under the EP should be limited to full beliefs, especially considering that experts tend to form numerical degrees of belief or credences in theories or propositions, e.g., 66–100% credence that the mean global temperature will increase by 1.5 degrees centigrade by the year 2052 (Hoegh-Guldberg et al., 2018).^2^ But Peterson appears to justify the restriction to qualitative, full beliefs in his account of the EP through the following remark,

If some quantitative (probabilistic) principle is adopted for reconciling divergent expert opinions, the policy maker may probably be presented with material that appears to be much more precise than it actually is. (2007, p. 9)

His remark seems to suggest that the trouble with a probabilistic representation of the EP is that the principle might be mistaken as a rule for aggregating conflicting expert opinions through some procedure like weighted averaging (Stone, 1961). Although aggregation rules may be convenient in summarizing diverging expert opinions, they risk overstating the evidence.

Peterson may have come to this conclusion by sharing a common intuition held by many about subjective probability, namely, that it is a *sharp* numerical representation of how strongly an individual (or group) believes something. It is the precise nature of probabilistic opinions that could cause decision-makers to be misled, and even more so if the probabilistic opinions of experts are combined, resulting merely in a precise summary statistic.

But Peterson’s reasoning is short-sighted considering that such a narrow conception of probability was challenged long ago by Frank Knight (1921) in his distinction between ‘risk’ and ‘uncertainty’. According to Knight, the former is subject to precise measurement, whereas the latter is not. Knight was not alone in taking issue with the precision of classical probability. Many others have also found alternative forms of mathematical probability to be desirable for epistemic and decision-theoretic reasons, including Isaac Levi (1974), Peter Walley (1991), and James Joyce (2010), for example.

In the next section, I will show that by adopting a broader conception of probability, namely, *imprecise probability*, there is a probabilistic representation of the doxastic commitments of decision-makers that is consistent with the EP, partially consistent with (OB)-(PER)-(PRO), and immune to the criticism that combined probabilities are likely to overstate the evidence.

## The Ecumenical Principle Represented by Imprecise Probability

Although full belief affords decision-makers convenience, less than full belief in scientific propositions may be inevitable due to uncertainty. Consider the novel coronavirus that has spread globally and affected over a hundred million people worldwide at the time of writing. Since medical researchers are still seeking an effective treatment for symptomatic patients, the proposition that treatment *t* effectively reduces symptoms (denoted as *Reduce*) remains uncertain, where *t* is in the domain of treatments currently under consideration.

In accommodating uncertain matters within an expert community, an expert’s (rational) degrees of belief or credences may be formally modeled by a probability function *p* on a (finite) Boolean algebra of propositions *B*, relative to a set of possible worlds *W*. But on a more liberal conception of subjective probability, an expert’s credences may instead be modeled by a non-empty set of probability functions ***P*** on the Boolean algebra. Credences under this more general mathematical representation are set-based rather than point-valued and can be imprecise.^3^

On occasions where an expert’s credences are imprecise, the imprecision might be due to the evidence made available at that time. Consider the COVID-19 pandemic, for example. Many things remain unknown at the moment, including which course of treatment is most effective for reducing symptoms, whether the newly developed vaccines provide immunity against different strains of the virus, how long face coverings should continue to be worn after a sizeable portion of the global population has been vaccinated, etc. Gaps in the evidence might compel an expert to form set-based credences in relevant propositions, e.g., ***P***(*Reduce*) = {0.5, 0.7} and ***P***(~ *Reduce*) = {0.3, 0.5}, and rationally so, as their credal state is fixed by the evidence in hand, no more and no less. The rationality of imprecise credences on occasions where evidence is incomplete but not absent ought to convince us that probabilistic representations should not be dismissed in characterizing the doxastic commitments of decision-makers under the EP.

But even in admitting that experts occasionally form rational credences and the credences may either be precise or imprecise, credences often differ from individual to individual, causing difficulty for decision-makers in forming judgments of their own. Consider, for example, a group of virology experts that agree on a class of antivirals to experimentally test on positive COVID-19 patients, but the virologists disagree about the effectiveness of each treatment individually, where credences are low, high, and somewhere in between across the set of possible outcomes. How should decision-makers go about taking into account the diverging expert credences? Classical probabilists might suggest employing an aggregation strategy such as *linear pooling*, where an expert’s opinion, for all propositions *X*, is weighted based on the expert’s reliability and combined through weighted averaging (Dietrich & List, 2016). However, it is this kind of procedure that leads to the issue Peterson is concerned about.

Consider a pair of medical experts, A and B, that have credences 0.3 and 0.9 in the proposition *Reduce*, respectively, and suppose that the experts are equally competent and reliable. The equal-weighted average in credence between them is 0.6. But as a collective or consensus opinion presented to decision-makers, it fails to convey the degree to which the two experts’ opinions diverge. Furthermore, 0.6 credence implies that collectively, the medical experts are more confident in *Reduce* than ~ *Reduce* under the weighted averaging rule, suggesting that *Reduce* has more evidential support when that is not the case. One can see now why Peterson is inclined to reject probabilistic principles for reconciling conflicting probabilistic judgments. His concern is further reinforced by the fair chance that the weighted averaging rule is the first aggregation strategy that comes to mind for many, given its familiarity.

But these observations of a specific kind of aggregation procedure should not convince us that all hope is lost for a feasible credal interpretation of the doxastic commitments of decision-makers under the EP. For linear pooling, of course, is not the only way to aggregate beliefs. Some have recently illustrated the epistemic advantages of alternatively aggregating beliefs through imprecise probabilities (see Stewart & Quintana, 2018). Drawing on the imprecise probability approach to belief aggregation and a recent account given by Elkin and Wheeler (2018) concerning peer disagreement, I propose that a decision-maker’s doxastic state is constrained by the following principle in case the views of experts diverge on a given matter.

IP. For all permissible doxastic states *D*, a decision-maker should adopt *D* if and only if *D* just consists of the union of expert opinions, $\bigcup_{i}\varvec{P}_{i}(X)$, for all *n* experts and propositions *X* in some Boolean algebra *B.*^4^

What this principle implies in the earlier case, for example, is that decision-makers should adopt imprecise credences ***P***(*Reduce*) = {0.3, 0.9} and ***P***(~ *Reduce*) = {0.1, 0.7} in light of the disagreement between medical experts A and B.

The proposed IP principle has several advantages. First, IP sidesteps an inconsistency that Peterson’s account gives rise to from his endorsement of the right to left direction of (PER)—if at least some experts believe *X*, then it is permissible to believe *X*. It follows from the right to left direction of (PER) that if the most influential experts believe *X*, then a decision-maker is permitted to believe *X*, despite some non-influential experts believing ~ *X*. But the EP prohibits decision-makers from ignoring or discarding the views of experts having less influence or prominence within the community. Hence the inconsistency. By comparison, IP does not satisfy the right to left direction (see below). Consequently, IP is less permissive. But failing to satisfy the right to left direction of (PER) is fortunate, not fatal, since the inconsistency is avoided, unlike on Peterson’s overly permissive qualitative account.

Second, IP is consistent with the EP as defined. It follows from IP that every expert view is taken into account under the required doxastic state *D*, as every expert view, ***P***_*i*_, is a subset of *D*. So, no expert’s view is ignored or discarded for lacking influence or prominence within the community. If, however, some expert’s view is not a subset of *D*, then, trivially, *D* does not consist of the union of all expert opinions for all propositions *X*, thereby violating IP. We see then that the general idea underlying the EP of accounting for every expert view is implied by IP, thus making the doxastic principle consistent with the EP.

Third, IP is partially consistent with Peterson’s constraints. On the (OB) requirement, IP satisfies the right to left direction. If all experts hold the same view, i.e., ***P***_1_ = … = ***P***_*n*_, then the required doxastic state *D* by IP is ***P*** such that ***P*** = ***P***_*i*_, for *i* = 1,…, *n*. IP, however, does not satisfy the left to right direction. Suppose that the required doxastic state *D* by IP is ***P***, but 1 and 2 differ in their views such that ***P***_1_ ≠ ***P***_2_. It is obviously false then that ***P*** = ***P***_*i*_, for* i* = 1,…, *n*.

On (PER), since IP implies that *D* is permissible if and only if *D* just consists of the union of all expert opinions for all propositions *X*, it logically follows that the views held by some experts are contained in *D*. IP, however, does not satisfy the right to left direction. But again, this is fortunate since IP avoids the inconsistency resulting from (PER) and the EP. To see how the right to left direction is not met, suppose ***P***_1_ is a subset of *D*, but ***P***_2_ is not, for experts *i* = 1, 2. The antecedent of the right to left direction of (PER) is true, but the consequent is false given that *D* does not consist of the union of all expert opinions for all propositions *X*.

On (PRO), IP satisfies the right to left direction provided that if the intersection of $\bigcup_{i}\varvec{P}_{i}$ and doxastic state *D* is empty, then *D* obviously does not consist of the union of all expert opinions for all propositions *X* and is impermissible. IP does not satisfy the left to right direction. Consider the above instance where ***P***_1_ is a subset of *D* but ***P***_2_ is not, for experts *i* = 1, 2. *D* is forbidden by IP, but the intersection of *D* and $\bigcup_{i}\varvec{P}_{i}$ is not empty. Thus, we find that IP only satisfies weaker versions of Peterson’s constraints. But violating the original constraints is quite reasonable, especially considering the inconsistency generated by (PER) and the EP.

Fourth, IP is immune to Peterson’s criticism against permitting probabilistic rules in characterizing the doxastic commitments of decision-makers under the EP. IP implies that a decision-maker’s doxastic state is fixed in accordance with the information in hand, no more and no less. In fact, a hallmark of imprecise probabilities generally is that they *match* the character of the evidence (Sturgeon, 2010). So much for overstating the evidence.

Finally, IP is more transparent than Peterson’s qualitative account on evidential support by realizing lower and upper bounds on credence. Consider the case of medical experts A and B from before. Under the IP principle, *Reduce* is given a minimum credence of 30% and a maximum credence of 90%. Decision-makers are thus committed to unique ranges of credence when conflicting evidence yields different levels of support for and against propositions. That is, IP commits decision-makers to adopting ranges of credence bounded by the least and greatest amounts of credence supported by their evidence.

The qualitative account, on the other hand, can mask the evidential support for some propositions. Unfortunately for Peterson, this exposes his view of the EP to an opposite concern. In particular, Peterson’s qualitative account is prone to *understating the evidence*. To see this, suppose that A categorically believes *Reduce*, whereas B categorically believes ~ *Reduce*. Decision-makers are not permitted to believe both propositions by logic alone, but they are entitled to choosing a side, given Peterson’s endorsement of (PER). Whichever side is taken, though, leads to a neglect of evidence since one of the experts’ opinions that is supposed to be recognized is completely discounted. The (PER) constraint consequently results in understating the evidential support that exists for the proposition(s) decision-makers choose to *disbelieve*.

Fortunately for IP, it safeguards against both exaggerating and neglecting evidence by stopping short of saying too much and saying too little. If precautionary-based reasoning prohibits both overstating and understating the evidence, then the IP proposal should be preferred to Peterson’s qualitative account in representing the doxastic commitments of decision-makers under the EP.

## Conclusion

I illustrated in this paper that the doxastic commitments of decision-makers under the EP can feasibly be interpreted probabilistically via the IP principle and that the representation is not subject to Peterson’s criticism against probabilistic rules. I also showed that the IP principle has further advantages, including avoiding an opposite concern that Peterson’s view is susceptible to, namely, understating the evidence.

While the proposed probabilistic account provides guidance on what to believe in the face of expert disagreement under precautionary-based considerations, I have not discussed in this paper how decision-makers should go about choosing among possible acts. Unfortunately, this issue is beyond the scope of the paper since the literature on decision-making with imprecise probabilities is vast and cannot be covered here. However, I will leave the reader with two suggestions that seemingly have a precautionary character. They are *maxi-min* (Gilboa & Schmeidler, 1989) and *maximality* (Walley, 1991).

The maxi-min rule requires decision-makers to maximize minimum expected value. The rationale behind the principle is that a decision-maker should err on the side of caution and select an option with the best worst-case outcome (see Wald, 1945). The maximality rule permits a decision-maker to choose an act if and only if that act maximizes expected value for all probability functions in the decision-maker’s set ***P***. In relation to IP, an act is permissible if and only if all experts view that act as maximal. Thus, IP and maximality imply some kind of unanimity decision criterion—that is, the views on which act(s) is best are unanimous for all experts. The upshot of maximality in this context is that the decision criterion is robust, giving decision-makers some reassurance that a permitted act is maximal by the lights of all experts.

Although both decision criteria appear to be precautionary in nature, neither is entirely uncontroversial. Again, it is beyond the scope of the paper to work out these details, but at least the two decision criteria offer some direction and furthermore illuminate the plausibility of IP since decision-makers are afforded more flexibility with respect to decision criteria when aiming to be precautious compared to a classical probabilistic representation and Peterson’s qualitative account.

## Footnotes

1 For further review, see Steel (2015) and Rechnitzer (2020).

2 It is important to note that full beliefs and degrees of belief or credences are not incommensurable. Peterson’s view may be consistent with the so-called Lockean Thesis (Foley, 1993), where an individual’s credence is sufficiently high enough to constitute outright belief in a proposition. For a recent account not only on the unification of full belief and credence but also their dynamics, see Leitgeb (2017).

3 Note that ‘imprecise probability’ encompasses more than sets of probability functions (see Walley (2000) for a review) and varies in motivation from evidential support (see e.g., Levi, 1974; Joyce, 2010) to ambiguity averse preferences (Gilboa & Schmeidler, 1989).

4 In disambiguating ‘opinion’ and ‘view’, I will treat the former in this context narrowly as the credence (precise or imprecise) given by an expert to a single proposition and the latter as the opinions of an expert with respect to all possibilities considered by the expert. Formally, an expert’s view is represented by a non-empty set of probability functions ***P*** on a Boolean algebra of propositions *B*, relative to a set of possible worlds *W*. Extended to *n* experts, a combination of views held by all experts consists of all the opinions of all possibilities considered by all *n* experts. Of course, this idealization assumes that there is agreement on the set of possibilities since all expert views are defined on the same Boolean algebra. Thanks to an anonymous referee for pointing out the ambiguity.
